# Rapid re-establishment of top-down control at a no-take artificial reef

**DOI:** 10.1007/s13280-022-01799-9

**Published:** 2022-11-02

**Authors:** Patrik Kraufvelin, Lena Bergström, Frida Sundqvist, Mats Ulmestrand, Håkan Wennhage, Andreas Wikström, Ulf Bergström

**Affiliations:** 1 Department of Aquatic Resources, Swedish University of Agricultural Sciences (SLU), Skolgatan 6, 742 42 Öregrund, Sweden; 2 Åland University of Applied Sciences, PB 1010, AX-22111 Mariehamn, Åland, Finland; 3 Department of Aquatic Resources, Institute of Coastal Research, Swedish University of Agricultural Sciences, Yttre Skällåkra 6, 432 65 Väröbacka, Sweden; 4 Department of Aquatic Resources, Havsfiskelaboratoriet, Swedish University of Agricultural Sciences, Turistgatan 5, Box 4, 453 30 Lysekil, Sweden

**Keywords:** Cod, Decapod crustaceans, Environmental compensation, Lobster, MPA, Restoration

## Abstract

Establishment of artificial reefs and no-take areas are management measures available for restoring deteriorated marine ecosystems, compensating for habitat loss and strengthening harvested populations. Following the establishment of no-take artificial reefs in western Sweden to compensate for hard bottoms lost to a shipping lane, we detected rapid positive effects on crustaceans and demersal fish compared to fished reference areas. The relative abundance and size structure of European lobster (*Homarus gammarus*) increased strongly in the no-take area indicating more than doubled and tripled egg production in 5 and 10 years, respectively. For benthic fish and crustacean communities, the abundances of gadoids and wrasses increased and the abundances of small decapod crustaceans decreased in the no-take area, likely indicating cascading effects of increased predation. The study demonstrates that relatively small no-take areas, enhanced by artificial reefs, can rapidly invigorate populations of lobster and fish that in turn may re-initiate local top-down control.

## Introduction

Human activities and their pressures are threatening the integrity and productivity of many marine environments and few un-impacted coastal areas remain globally; with pristine marine ecosystems being especially rare in Europe (Korpinen et al. 2021; Williams et al. 2022). Coastal development may cause loss and disturbance of highly productive shallow habitats, while overharvesting of marine living resources may deplete populations of commercially and ecologically important species (Airoldi and Beck 2007). This impairs ecosystem functions and important ecosystem services from large predatory species, such as biological regulation through top-down control, which may entail a mesopredator release and a weakened grazing upon ephemeral algae (Östman et al. 2016). To reverse and counteract similar negative trends, a suite of conservation, prevention, mitigation, restoration and compensation measures can be applied (Duarte et al. 2020; Moland et al. 2021). One way to compensate for loss and disturbance of hard bottom habitats may be to construct artificial reefs (Seaman 2007). No-take marine reserves, where no fishing is allowed, have in turn proven effective for protecting habitats and invigorating overharvested species (Fenberg et al. 2012; Sciberras et al. 2013; Moland et al. 2021; Knutsen et al. 2022). Herewith, the EU biodiversity strategy contains ambitious commitments for nature protection by aiming to extend the network of protected sea areas within EU to 30% and ensuring that at least 1/3 of these are strictly protected (European Commission 2020; Roberts et al. 2020). A combined measure of marine protection and reef establishment might be particularly effective, especially as no-take areas that include reefs seem to generate greater positive effects than areas devoid of reefs (Lester et al. 2009).

The Vinga artificial reefs were constructed in 2003–2004 in the archipelago of Gothenburg in northern Kattegat on the Swedish west coast, at the transition between the Baltic Sea and the northeastern North Sea (Skagerrak). A cluster of seven artificial reefs, also protected from fishing through Swedish national fisheries regulations (FIFS 2004:36, see https://www.havochvatten.se/vagledning-foreskrifter-och-lagar/foreskrifter.html), was established with a primary focus on creating habitats for European lobster (*Homarus gammarus*) and to compensate for a loss of natural reef habitats in connection with deepening and widening the main shipping route to Gothenburg harbor. Due to the high fishing pressure in the region at this time (Svedäng and Bardon 2003; Sundelöf et al. 2013; Moland et al. 2013b), the additional implementation of a no-take area was expected to benefit also other local populations than lobster, particularly large predatory fish such as cod (*Gadus morhua*). In the region, lobster has experienced a long-term decline due to fishing since the 1940s and was at the time of the reef establishment at historically low levels (Moland et al. 2010; Sundelöf et al. 2013). Fish assemblages are strongly affected by fishing in the area, and subpopulations of gadoids have declined severely since the early 1980s (Svedäng and Bardon 2003), although the populations are also affected by decreased availability and poorer conditions of essential habitats (Bergström et al. 2016; Kraufvelin et al. 2018).

Total fishing closures are effective measures to restore the abundance and size structure of target species of fisheries and to enhance the diversity of local biota (Halpern 2003; Fenberg et al. 2012; Moland et al. 2021). Evidence suggests that also quite small no-take areas can produce significant biological responses (Lester et al. 2009; Moland et al. 2013b; Bergström et al. 2019). As both commercial and recreational fisheries often target large predatory species, an increase in these species may also indirectly affect other parts of the ecosystem through effects of their structuring role in the food web (Guidetti 2006). More predators decrease the abundance of prey species, enforcing top-down control of the local food web, which may sometimes cascade all the way down to the level of primary producers, including habitat-forming species (*cf* Östman et al. 2016; Bergström et al. 2019). No-take areas may also influence adjacent areas and systems positively through spillover effects of adult fish and export of pelagic eggs and larvae (Abesamis and Russ 2005). The extent of these effects vary between taxonomic groups and geographical areas, however, and not all targeted species respond positively to protection (Gill et al. 2017; Giakoumi et al. 2018).

The use of artificial reefs, here defined as submerged structures deployed on the seabed to mimic some characteristics of natural reefs, represents another management measure that is becoming increasingly widespread in marine environments (Seaman 2007). By contributing with three-dimensional firm structures, artificial reefs form new habitats and increase the overall complexity of the seascape. The reefs offer suitable substrates for colonization by benthic and epiphytic macroalgae and sessile animals that may in turn enhance populations of small motile species and juveniles locally by providing additional food resources and shelter from predators (Pickering and Whitmarsh 1997). Through these mechanisms, the artificial reefs may also benefit target species for fisheries, such as the European lobster and large predatory fish. Artificial reefs are used for both fisheries enhancement and ecosystem restoration, including environmental compensation, mitigation of environmental impacts and habitat management (Claudet and Pelletier 2004; Levrel et al. 2012). Similar responses are also documented for so-called secondary artificial reefs (Pickering et al. 1998), which are constructions introduced for other purposes such as bridges and piers, oil platforms, foundations for wave energy converters and wind energy turbines (Bergström et al. 2013; Dannheim et al. 2019).

This study evaluates the Vinga no-take artificial reefs (hereafter NTAR) and their roles for invigorating crustacean assemblages and demersal fish, first based on yearly sampling after their establishment and then with follow-ups 11–12 years later, to examine long-term effects. The study focuses primarily on changes in the abundance and size of dominating species and in assemblage composition by comparing the NTAR to nearby fished areas (hereafter reference area). In addition, developments over time in prey species, mainly small crustaceans, are assessed as indications of changes in trophic control. Hence, the study evaluates several original objectives for establishing the Vinga NTAR (Egriell et al. 2007) such as to:

I. increase the abundance and mean size of lobster within the NTAR to invigorate the overall population through export of larvae and adult migration to adjacent fished reference areas (spillover),
II. provide an effective protection of gadoids in the NTAR with special consideration to the function of artificial reefs as foraging areas and refuges, and
III. re-establish the top-down control of the fish and crustacean assemblages, a central ecosystem function upheld by lobster and large predatory fish.

## Materials and methods

### Study area

**Fig. 1:**
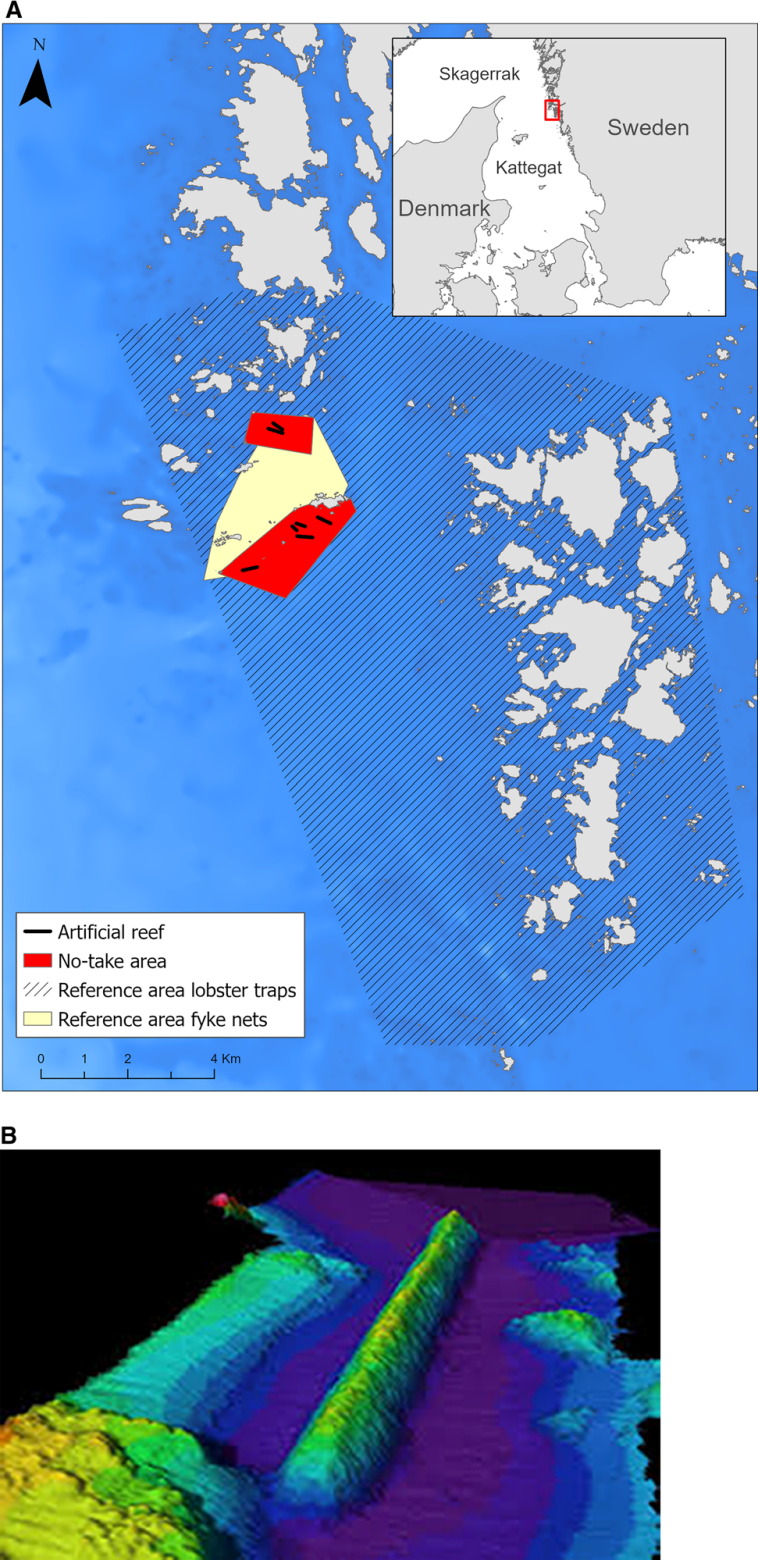
**A** The NTAR represented by red polygons at Vinga outside Gothenburg, Sweden. The black bolded lines represent the locations of artificial reefs. The yellow polygon represents the location of fyke net sampling stations. Fyke net stations outside the NTAR (the red polygons) constitute the reference, located in the area open to fishing. For the targeted lobster sampling, reference data were additionally collected from a geographically more widespread area by collaboration with local fishers (the striped polygon). **B** A three-dimensional multibeam sonar image of reef 1A-1 Buskär at roughly 26–28 m depth. This reef is 350 m long, 45 m wide and 14 m high (above bottom) with its highest point 13.5 m below the surface. The image is reproduced with permission from the Swedish Maritime Association

The Vinga artificial reefs constitute a cluster of seven ridges made of blasted rock, which were deployed on sandy bottoms at 20–37 m depth 2003–2004. The ridges are 130–380 m long, 30–45 m wide and 4–14 m high, with 12–25 m of water above (Egriell et al. 2007; Fig. 1A, B). A total volume of rock of 800,000 m^3^ was used for creating the reefs. This material originated from the deepening and widening of the shipping route to Gothenburg. Two no-take areas were established around the artificial reefs; Tanneskär (1.2 km^2^) in the north surrounds two created ridges and Buskär (3.2 km^2^) in the south surrounds the remaining five ridges (Fig. 1A). Due to the areas’ close proximity and their similarities with regard to design and construction history, their connectivity is assumingly high and thus we consider the two areas making up the NTAR as one. The reference areas are open to fishing, but subject to the same general fishing regulations as most other parts of the Swedish Kattegat/Skagerrak coast, including catch regulations of lobster and cod and a total ban of bottom trawling.

### Field studies

One objective of the field studies was to document the colonization of lobsters within the NTAR to the artificial reefs and natural lobster habitats (hard bottoms) and we did this using lobster traps. However, we did not sample with lobster traps in reference areas outside the NTAR, where lobster fishing is normally allowed. To compare the lobster abundance in traps between the NTAR and the reference area, we used instead catch information from lobster fishers. This included information from one recreational fisher with 28 lobster traps active in the area close to the NTAR and information from a professional fisher with 50 lobster traps fishing in a wider area outside the NTAR (these reference areas are visible in Fig. 1A). These fishers fished in the same way over the years as we did in the project monitoring inside the NTAR (see below).

We monitored the temporal development of lobster using lobster traps during October–November 2002–2006, 2008–2010 and in July 2014. In total, each year, we set 20 traps by four of the artificial reefs at 12–18 m depth and additionally 20 at natural hard bottoms at 12–20 m depth (Fig. 1A). The ambition was to check and reset the lobster traps twice weekly during monitoring periods. However, due to weather conditions, average soak time was instead 5.2 days, with a minimum of 2 days and a maximum of 20 days. We hauled each trap for an average of 8.4 times per year/season. We registered all lobsters in the catches, together with information on their sex and carapace length. We subsequently tagged the lobsters with Floy T-bar anchor tags attached to the side of the abdominal segment and released the tagged lobsters at the capture location. In the analyses, we estimated catch per unit effort (CPUE) as the number of lobster per trap and fishing day. Half of the traps in each area were of a circular type (so called Swedish model, 120 cm long with two entrances, one chamber and no escape openings) and half were of the semi-circular Scottish model (90 cm long). We randomized the trap model at each sampling site. All traps had a mesh size of 50 mm and we baited the traps with salted flounder or herring.

Fyke net fishing targeting the benthic community of fish and decapod crustaceans took place both inside and outside the NTAR with stations grouped around the artificial reefs in the NTAR and distributed randomly in the reference area (Table 1). The fyke nets were 55 cm high, with a semi-circular opening, a 5 m long arm and a mesh size of 10 mm, which corresponds to the national monitoring standard in Sweden (Leonardsson et al. 2017). We placed the fyke nets at randomly distributed stations close to the reefs in the NTAR, and in the reference area (Fig. 1A). We fished each station for approximately 20 h (over one night), using 5–6 connected fyke nets per station. We estimated CPUE for each station, as the number of fish per fyke net and fishing night. Thus, since the number of fyke nets per station varied somewhat, CPUE was standardized by the number used. Hence, the survey plan encompassed 30 independent stations annually during 2002–2006 and then again in 2015, in the NTAR and reference area, respectively, to some extent covering the time before the AR were established. In 2002, before the reefs were in place, we distributed the fished stations around the planned reef locations. In autumn 2003, approximately half of the reefs were constructed, and we fished at stations positioned by the reefs. From 2004 onwards, we distributed the fished stations randomly around the completed reefs, based on positions on the sea chart. We then kept the same stations during subsequent years (Fig. 1A). During this time, the final number of samples per year varied between 18 and 30 (on average 26 stations) per area, as we could not obtain data from all planned 30 stations every year due to weather conditions.

**Table 1:** Characterization of surveys and data sets used in this study in relation to the objectives of the establishment of the NTAR

Objective	Survey	Years	Target questions and statistical analyses
I. To increase the abundance and mean size of lobster within the NTAR to invigorate the overall population through export of larvae and adult migration to adjacent fished reference areas (spillover)	Lobster trap survey; Parallel sampling on artificial reefs (no-take, AR) and natural habitats (no-take, natural habitat) inside the NTAR Additional data from a nearby reference area (fished, natural habitat) provided by local recreational and commercial fishers (the striped polygon in Fig. 1A) Tagging and release of lobster individuals at their capture location to determine spillover through re-catches	2003–2006, 2008–2010, 2014 for lobster trap monitoring within the NTAR; 2003, 2005, 2007–2014 for additional data in the nearby reference area	Comparison of the development of catches at artificial reefs and natural hard bottoms in the NTAR (general linear model, GLM) Comparison of the development of catches inside the NTAR and in the nearby reference area (GLM) Following the size development of lobster inside the NTAR (linear regression) Estimation of changes in egg production from changes in lobster abundance and mean female size Evaluation of spillover effects by registration of recaptures of tagged lobster inside and outside the NTAR
II. To provide an effective protection of gadoids in the NTAR with special consideration to the function of artificial reefs as foraging areas and refuges	Fyke nets; Parallel sampling in the NTAR (combined over artificial reefs and natural habitats) and in the nearby reference area (fished, natural habitat)	2002–2006 and 2015	Comparison of the development in species composition of demersal fish assemblages (including decapod crustaceans) between the NTAR and the reference area over time (PCO, two-way PERMANOVA) Comparison of the catch development of individual fish and crustaceans, including changes in size of cod and lobster, inside the NTAR and reference area over time (GLM)
III. To re-establish the top-down control of the fish and crustacean assemblages, a central ecosystem function upheld by lobster and large predatory fish	Same as II	Same as II	Comparison of the development of species within different trophic groups over time in the NTAR and in the reference area (linear regression)

Each year, we fished with fyke nets during 6–9 days in October. We registered catches as number of individuals per fish/crustacean species, and we noted individual lengths to the closest cm. We located the fyke net stations at 18–28 m depth in the NTAR and at 20–27 m depth in the reference area. Based on measurements taken in connection with the sampling, the average water temperature ranged between 10.1 and 15.1 °C during the sampling periods, with similar temporal patterns in both areas, whereas the salinity range was 30.1–31.7.

With regard to trophic level, we categorized the species caught in fyke nets into large predators, mesopredators and prey, based on their feeding habits and using trophic values from Froese and Pauly (2015). We grouped lobsters as large predators together with the fish species known to feed on fish and crustaceans, i.e. cod and poor cod. We classified the remaining fish species as mesopredators, whereas we categorized spider crabs, swimming crabs, hermit crabs and shore crabs as prey (Ennis 1973; Armstrong 1982; Pihl and Wennhage 2002; Floeter and Temming 2003), however considering that mesopredatory wrasses can prey on juvenile crabs (Sayer et al. 1996).

The samplings complied with ethical standards under permission from the Swedish Authorities. We followed all applicable international, national and institutional guidelines for the care and use of animals.

### Statistical analyses

We assessed catch data from lobster traps for changes over time using general linear models, GLM, with the factor *Area* and the continuous variable *Year.* We used the interactions between these as tests for differences between areas in the relative development of lobster abundance over time. We ran separate assessments to compare (i) artificial reef habitats with natural habitats inside the NTAR, (ii) artificial reef habitats in the NTAR with natural habitats in the reference areas, as well as (iii) natural habitats in the NTAR with natural habitats in the reference areas. Additionally, we tested for potential changes in the size of caught female and male lobsters over time in the NTAR by linear regression. We subsequently estimated potential changes in lobster egg production in the NTAR over time from registered changes in lobster abundance and mean female size assuming a linear relationship between female size and number of eggs (Agnalt 2008).

For fyke nets, we analyzed changes in overall species composition over time during 2002–2006, with 2015 as an additional sampling year, by multivariate Principal Coordinates analysis (PCO) and two-way PERMANOVA. For visual clarity, we aggregated the data for the PCO to average abundances per species and year, separately for the NTAR and the reference area. We quantified similarity in species composition among all samples by the Bray–Curtis similarity index, after square-root transformation to balance the relative influence between dominating and rare species. We disclosed pelagic fishes and small crustaceans as these are not caught representatively by fyke nets. We identified species contributing most to differences among areas primarily based on their vector loadings on the first PCO axis. We tested for significant differences in the development of species composition between the two areas over the entire set of years, 2002–2006 and 2015, using two-way PERMANOVA with the fixed factor *Area* (two levels: NTAR and reference area) and the random factor *Year* (with six levels) and with individual fyke net samples as replicates.

We subsequently assessed the temporal development of the most influential species, identified by the multivariate analysis, by univariate analyses. This concerns European lobster, cod, poor cod, rock cook (*Centrolabrus exoletus*), corkwing wrasse (*Symphodus melops*), shore crab (*Carcinus maenas*), spider crabs (*Hyas* spp.), swimming crabs (Portunidae) and edible crab (*Cancer pagurus*). Additionally, we evaluated the temporal development in cod lengths, in the abundance of large cod (> 30 cm length) and in large lobster (> 23 cm body length/81.6 mm in carapace length). Similarly to the evaluation of lobster trap data, we tested for differences in the development in abundance of these species using a GLM with the factor *Area* and the continuous variable *Year* (2002–2006 and 2015), where a significant interaction between the two variables indicated different developments between the areas.

We assessed relationships between species representing different trophic levels, i.e. total abundance of predators, mesopredators and prey, respectively, by pairwise linear regressions after ln (*x* + 1) transformation of each variable. We included all available data pairs from fyke net fishing and we ran the analyses separately for the NTAR and the reference area.

We ran multivariate analyses with PRIMER PERMANOVA 7 (Clarke and Gorley 2006; Anderson et al. 2008) and univariate analyses using IBM SPSS 26. Before running parametric univariate tests, we checked for normality by Kolmogorov–Smirnov’s test and homogeneity of variances by Levene’s test and applied transformations in case assumptions were not met.

## Results

### European lobster

**Fig. 2:**
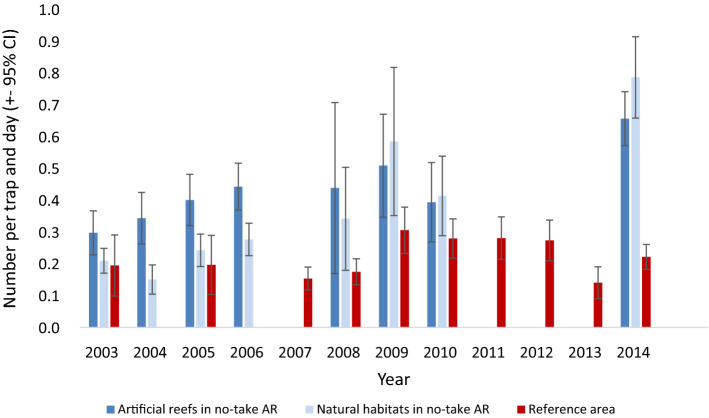
Number of lobster per trap and day (CPUE) at the artificial reefs (dark blue) and at the natural lobster habitats (light blue) within the NTAR in 2003–2006, 2008–2010 and 2014, and in the reference area until 2014 (red). Error bars show ± 95% confidence intervals. Note that the zeros for different years in the table represent missing data and not zero-catches

Lobster catches in traps increased more over time inside the NTAR compared with the reference areas, both for the no-take artificial reefs and no-take natural habitats (GLM, Site × Year interactions *p* < 0.001; Fig. 2, Table 2). Based on fitted regression lines, lobster densities increased on average with 198% in the NTAR between 2003 and 2014, as compared with 22% in the reference area. In 2014, average lobster catches inside the NTAR were about 3–3.5 times higher than in the reference area, compared with roughly equal values in 2003 when the NTAR was established (Fig. 2). Comparing natural habitats with artificial habitats inside the NTAR, the lobster abundances increased faster at the artificial reefs during the first years, which also is evident from a significant Site × Year interaction (Table 2). However, these differences were no longer evident from 2008 onwards (Fig. 2).

**Table 2:** Interaction effects between Site × Year from GLMs assessing differences in the temporal development of lobster abundances in traps (CPUE) between different areas. The analyses were run separately to compare artificial reefs with natural habitats inside the NTAR, artificial reefs in the NTAR with the reference area as well as natural habitats in the NTAR with the reference area. Years cover the period 2003–2014, for details see Table 1

Test	*F*	*p* value
Artificial reef habitats vs natural habitats in the NTAR	*F*_1,239_ = 12.74	< 0.001
Artificial reef habitats in the NTAR vs natural habitats in the reference (fished) areas	*F*_1,445_ = 11.72	< 0.001
Natural habitats in the NTAR vs natural habitats in the reference (fished) areas	*F*_1,444_ = 67.35	< 0.001

The mean size of both male and female lobster increased in the NTAR after its establishment. In 2003, the mean carapace length was ca 80 mm for females and 81 mm for males, reaching 93 mm for females and 95 mm for males in 2014. Regression analyses indicate a slightly steeper increase in average size over time for males (*y* = 83.84 + 1.26*x*, *R*^2^ = 0.79, *F*_1,6_ = 22.1, *p* = 0.003) than for females (*y* = 79.98 + 0.96*x*, *R*^2^ = 0.81, *F*_1,6_ = 26.0, *p* = 0.002). Together, the increase in lobster density and size clearly indicate a higher lobster biomass in the NTAR since its establishment. The increases in density and female size also imply a larger egg production, increasing with 29.6% in 2005, 46.6% in 2006, 63.5% in 2008, 129.2% in 2009 and 229.0% in 2014 compared with values 2003–2004.

Results from the tagging of lobsters caught in traps (2225 individuals tagged in total) showed that of 1540 recaptures until 2009, 93.2% were caught within the NTAR (i.e. sampled within this study, Fig. 1A) and only 6.8% were reported from commercial/recreational fisheries outside the NTAR. The rate of migration between the two parts of the NTAR (situated approximately 2 km apart; see Fig. 1A) was estimated to 0.5% for Tanneskär in the north and 1.9% for Buskär in the south, based on our recaptures. While most lobsters caught outside of the NTAR were recaptured within 0–10 km, one lobster migrated as far as 80 km to the south and another lobster 60 km to the north.

### Fish and crustacean assemblages

In all, we registered 29 fish species in the fyke net surveys. Of these, we caught 24 in the NTAR and 27 in the reference area. Of nine species of decapod crustaceans in total, we caught seven species in the NTAR and all nine in the reference area.

**Fig. 3:**
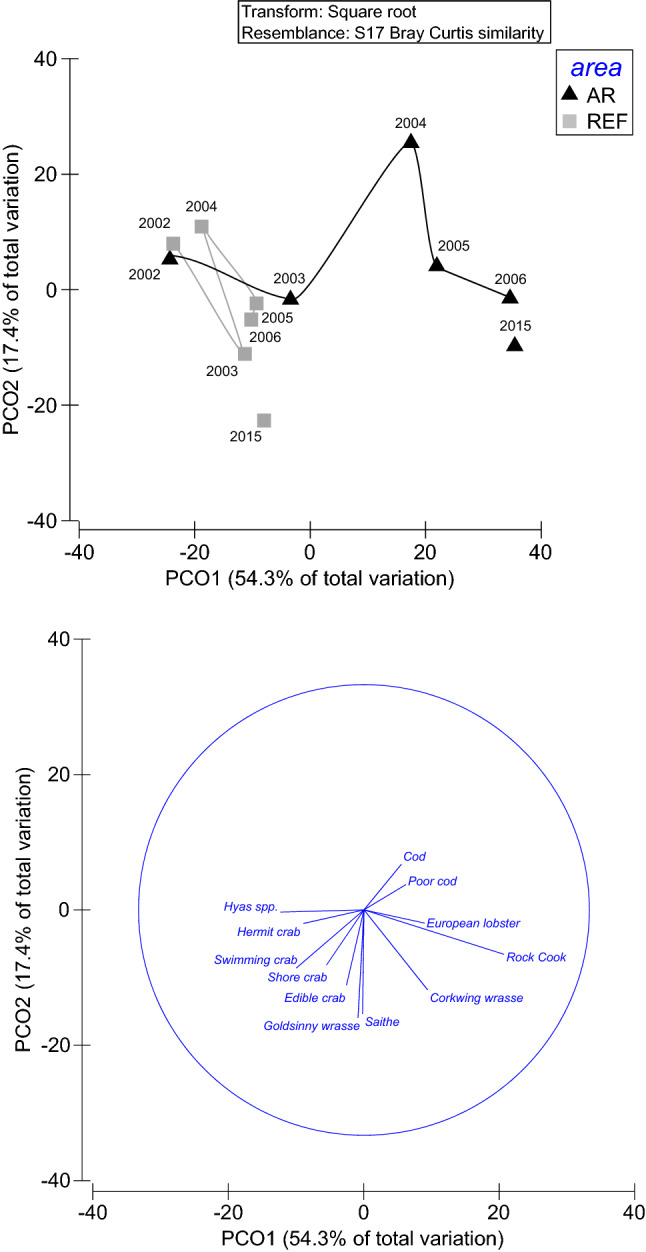
Results from PCO analysis showing similarity in species composition between the NTAR (AR in the figure) and the reference area (REF in the figure) during 2002–2006 and in 2015 (above) together with vector overlays demonstrating species responsible for differences (below). Similarity was quantified by the Bray–Curtis index on square-root transformed data. The NTAR was established in 2003

Multivariate PCO-analyses showed that the species composition was very similar in the NTAR and in the reference area in 2002 in the beginning. However, after the reefs and the no-take area were established in 2003, the species composition in the NTAR rapidly diverged from that of the reference area (Fig. 3). The overall divergence was reflected in a significant interaction between the factors *Area* and *Year* according to a two-way PERMANOVA using all fyke net fishing stations as replicates (Pseudo-*F*_5,301_ = 3.84, *p* < 0.001). Individual pair-wise tests during different years revealed no significant differences in species composition before the NTAR had been fully established, while differences between areas became highly significant (p < 0.001) from 2004 onwards. The different trajectories in species composition in the two areas over time were primarily reflected along PCO1, which encompassed 54.3% of the total variation in the data. Among the species contributing to changes along PCO1, we classified cod, poor cod and European lobster as large predators (and these had all positive PCO1-loadings of 0.25, 0.22 and 0.23, respectively), while we classified rook cook and corkwing wrasse as mesopredators (also with positive PCO1-loadings of 0.55 and 0.26, respectively). We found negative PCO1-loadings for potential prey species: spider crabs (PCO1-loading − 0.45), swimming crabs (− 0.30), hermit crabs (− 0.29) and shore crabs (− 0.15). Hence, all species with positive PCO1-loadings were predators/mesopredatory fish, while all species with negative loadings were potential prey species (mesopredatory crabs).

**Fig. 4:**
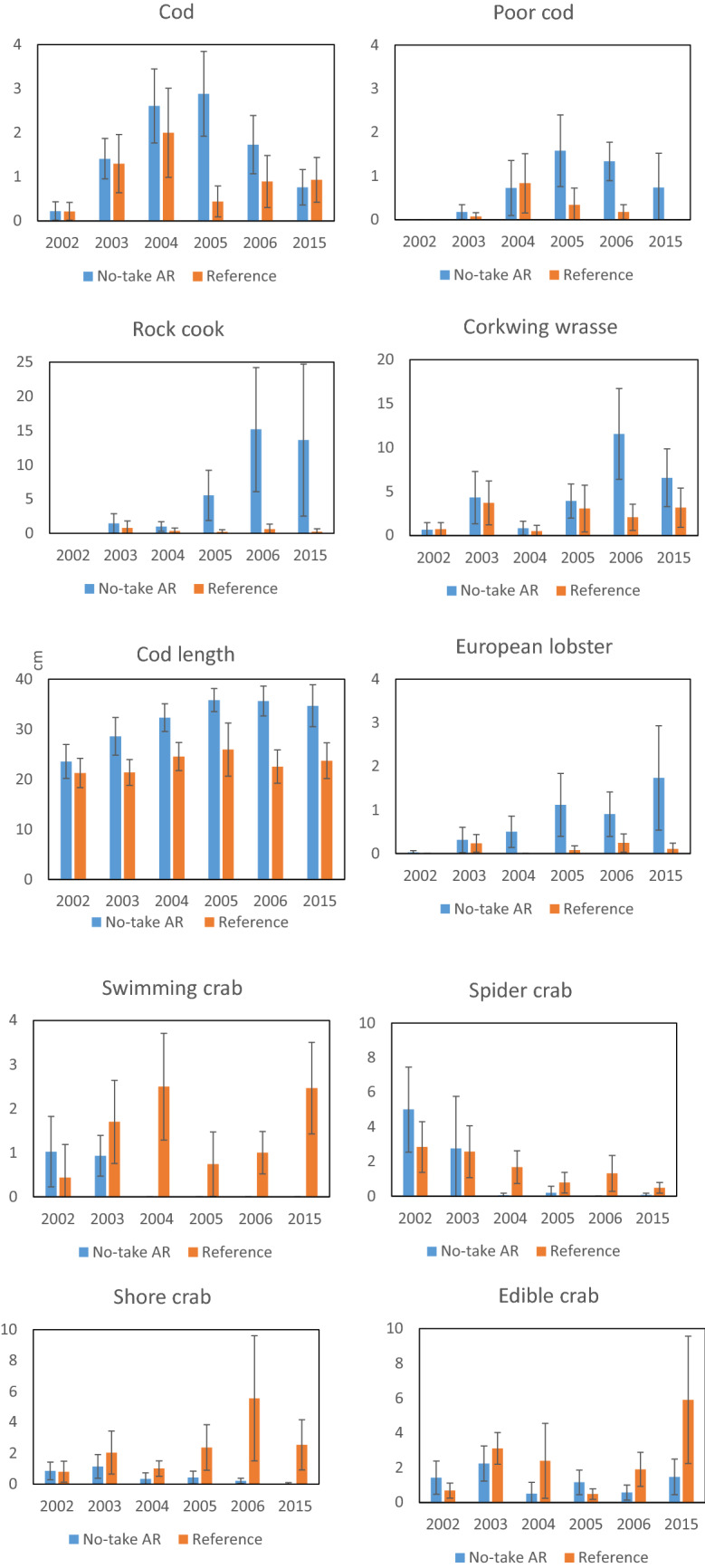
Fyke net catches (mean abundance per station and night ± 95% confidence intervals) of predatory fish, mesopredatory fish and decapod crustaceans as well as cod length in the NTAR and in the reference area 2002–2006 + 2015 (all zero values represent no individuals caught)

Univariate analyses of the development of individual species in the NTAR and in the reference area 2002–2006 (Fig. 4, Table 3) verified that catches of the most common fish species had different developments over time in the NTAR and the reference area. The relative abundance of all these species increased over time in the NTAR, at least until 2006, while the abundance of smaller decapod crustaceans decreased (Table 3, left columns). The fyke net surveys also showed an increase in lobster over time in the NTAR, hence supporting the results from the trap survey. Compared with when the NTAR was established in 2003, lobster catches in fyke nets were > 3 times higher in 2006 and almost six times higher in 2015 and by this time, 17 times more lobster were found in fyke nets from inside the NTAR than in the reference area (Fig. 4). Looking at long-term effects, the previous increases in cod and poor cod catches, however, had disappeared by 2015. A similar pattern was also evident for large cod (abundance of cod > 30 cm in length and mean cod length; Fig. 4, Table 3, right columns). For the swimming crab, spider crab and shore crab, there were significant decreases in the NTAR over time, whereas for the edible crab, this change was most apparent during the revisit in 2015 (Fig. 4, Table 3).

**Table 3:** Interaction effects between Site × Year from GLMs assessing differences in the temporal development of individual species between the NTAR and the reference area, based on the fyke net survey. Results are shown separately for years 2002–2006 as well as for longer term effects with year 2015 added

Response variable	GLM 2002–2006		GLM with 2015 added	
	*F*_1,249_	*p* value	*F*_1,309_	*p* value
Rock cook	35.37	** < 0.001**	21.43	** < 0.001**
Corkwing wrasse	20.27	** < 0.001**	4.67	**0.031**
Poor cod	19.96	** < 0.001**	2.42	0.121 ns
Cod	8.02	**0.005**	1.35	0.245 ns
European lobster	8.79	**0.003**	11.38	** < 0.001**
Swimming crab	9.92	**0.002**	21.90	** < 0.001**
Spider crab	5.20	**0.023**	0.33	0.564 ns
Shore crab	20.57	** < 0.001**	6.55	**0.011**
Edible crab	0.86	0.356	6.19	**0.010**
Cod > 30 cm	16.25	** < 0.001**	0.08	0.776 ns
Lobster > 23 cm	14.63	** < 0.001**	12.87	** < 0.001**

Response variable	GLM 2002–2006		GLM with 2015 added	
	*F*_1,333_	*p* value	*F*_1,384_	*p* value
Cod length	4.92	**0.027**	1.24	0.266 ns

### Trophic relationships inside and outside the NTAR

**Fig. 5:**
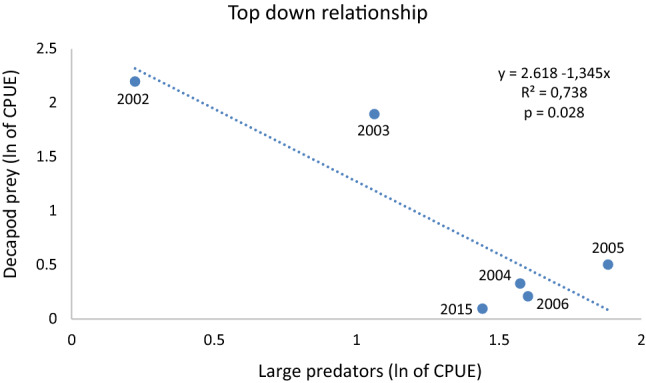
Relationship between the relative abundance of decapod prey species in relation to large predators (sum of lobster, cod and poor cod, i.e. species known to feed on crustaceans) in the NTAR during the years 2002–2006 and 2015. The equation shows results from a linear regression analysis (df = 1, 4) on ln (*x* + 1) transformed CPUE data (catch per unit effort)

Plotting trophic relationships between the abundance of potential prey and potential predators over time and linear regression analysis rendered one interesting result. This was the significant negative relationship (*p* = 0.028) between decapod prey species and the total abundance of large predatory species (lobster, cod and poor cod) indicating an increased (re-established) top-down control over time in the NTAR (Fig. 5). The relationship between the total abundance of decapod prey species and mesopredatory fish species, i.e. rock cook and corkwing wrasse was non-significant in the NTAR (*p* = 0.155). Corresponding analyses for the reference area showed no significant relationships for decapod prey species plotted against the total abundance of large predatory species (*p* = 0.103), nor when plotted against the total abundance of mesopredatory fish species (*p* = 0.687).

## Discussion

The Vinga NTAR exemplifies that establishment of small marine protected areas in combination with artificial reefs can contribute to distinct positive effects in terms of increasing abundances of target species for fisheries and re-established top-down control of lower trophic levels. Clearly higher abundance and larger size of lobster were evident in the NTAR compared with the reference area within a few years after implementation, and persisted over the 12-year time span of the study. Combined, the increased density and size of female lobsters implied a more than a doubled and tripled egg production in five and ten years, respectively. For gadoids, the abundances were higher in the NTAR during the first four years after establishment, but these differences disappeared over the longer period. Other observed effects were an increase in the abundances of wrasses as well as a decrease in abundances of smaller decapod crustaceans. The latter are common prey for larger fish and lobster. Since most of the species favored by the NTAR are predators that are capable of controlling ecosystem structure and function, the results likely mirror a re-established top-down control in the protected area.

Revisiting the objectives of the NTAR-establishment (see end of Introduction); some were met, while others were met only partly. For objective I, both the abundance and the mean size of lobster increased over time within the NTAR, while there were few signs of spillover effects based on recaptures of tagged individuals outside the areas (Moland et al. 2011, Thorbjørnsen et al. 2018; Nillos Kleiven et al. 2019). Lobster larval production and export, on the other hand, could have increased substantially because of the increase in female spawning biomass and egg production over time. For objective II, gadoids increased in the NTAR compared with the reference area until 2006, but there was no significant difference between the two areas in 2015. For objective III, there were indications of a re-established top-down regulation of small crustaceans by lobster and large predatory fish in the NTAR.

The observations of increased densities and sizes of lobster inside the NTAR are supported by other studies from the northeast Atlantic (e.g. Moland et al. 2011, 2013a, b, Thorbjørnsen et al. 2018; Nillos Kleiven et al. 2019). In our traps, lobster density showed a yearly increase of 10.4% in the NTAR and 1.8% in the reference area between 2003 and 2014. Here, it should be noted, however, that the reference data stemmed from professional and recreational fishers and was not directly part of the sampling program of this study, although the same fishing methods were applied in both areas. It should also be noted, that the lobster trap data from 2014 in the MPA were from July, while the reference area was fished normally in October this year as in all other years. A close check of data taken both in July and in October in the MPA during other years gives, however, no indications of crucial differences in lobster abundance and size between July and October. Additionally, the sometimes highly variable soaking times for the lobster traps (due to weather conditions) could cause some problems with the CPUE, although there were no systematic differences in soaking times and potential errors due to this are rather part of the natural variability than ones imposing impact on the analyses and their interpretations. In comparison with our data above, Moland et al. (2013b) registered an average increase of 6.6% per year over 14 years at a 2.2 km^2^ lobster reserve at Kåvra, Sweden. On the other hand, Moland et al. (2013a) reported an abundance increase of 245% in protected areas over four years (36% yearly increase on average) compared with an abundance increase of 87% in reference areas (17% yearly increase on average) along the Norwegian Skagerrak coast.

The higher lobster abundance on the artificial reefs compared with the natural habitats in 2004–2006 probably reflected a stronger initial attraction to the artificial reefs during the first years after the establishment of the NTAR (Fig. 2). From autumn 2008 on, this difference was no longer evident, which may be explained by that the available AR habitats had been filled up, or that the further increase in lobster abundance hereafter was explained by the absence of fishing. Previous studies in the Kåvra reserve showed a similar development (Moland et al. 2013b), supporting this finding. For our fyke net fishing, data did not allow to separate between effects of the habitat (artificial versus natural reefs) and fishery regulation (establishment of no-take areas).

At the Vinga NTAR, we estimated the average length of lobsters to increase with 1.55% per year over 11 years. These results are similar to the ones from the Kåvra reserve where lobster lengths increased with 1.6% per year over 14 years (Moland et al. 2013b), but clearly lower than in protected areas along the Norwegian Skagerrak coast, where lobster lengths increased with 3.2% per year over four years (Moland et al. 2013a). In Lamlash Bay, Firth of Clyde, Scotland, four-year surveys revealed 110% greater lobster catches per unit effort, 190% greater weight per unit effort and 10–15 mm greater carapace lengths within the no-take reserve compared with reference areas (Howarth et al. 2017). Similarly, studies at Lundy Island, Bristol Channel, England, provided evidence of a rapid, large increase in the abundance and sizes of lobsters within a no-take zone (Hoskin et al. 2011; Davies et al. 2015). As larger size of female lobsters leads to more and larger eggs and larger larval size at hatching (Agnalt 2008; Moland et al. 2010), the increases in abundance and size of lobster may have clearly positive implications for local lobster populations. In the current study, we estimate the lobster egg production in the NTAR to have more than doubled in 5 years (2009) and more than tripled in 10 years (2014) compared with 2003–2004.

Another study from the Kåvra reserve showed adult lobsters to be highly stationary (Øresland and Ulmestrand 2013) and similar results have been shown from the Norwegian Skagerrak coast (Moland et al. 2011, Thorbjørnsen et al. 2018). Out of > 4000 tagged lobster individuals, for instance, only 1.4% were recaptured outside the 2.2 km^2^-protected area in Kåvra (Øresland and Ulmestrand 2013). In the study from the Vinga NTAR, the proportion of adult lobsters migrating out from the NTAR was higher, 6.8%, even though the results still indicate a restricted adult migration and high level of site fidelity. It is noteworthy, however, that specimens recaptured by traps outside the NTAR, may be underestimated as these recaptures were estimated from reports by professional and recreational fishers, i.e. it took place outside the survey program of the study. Still, the estimated increased egg/larval production from more and larger lobsters may also have contributed positively to lobster populations outside the NTAR.

Effects of the NTAR on the assemblages of fish and decapod crustaceans were not as consistent as for lobster, even though we can see some clear patterns. There were evident differences between trophic guilds, with predators and mesopredators increasing in the NTAR and prey species increasing in the reference area (Fig. 3). For both the NTAR and the reference area, species compositions during the follow-up study in 2015 appeared quite similar to the ones in the same areas nine years before, in 2006. This indicates that the rapid changes observed in during the initial years could have ended as early as after 3–4 years (in 2006), whereafter the assemblages might have stabilized at a new level. With regard to the dominating piscivores, both cod and poor cod increased in the NTAR during the initial years 2002–2006, and we could see a parallel increase in the abundance of large cod individuals as well as in the mean length of cod (Table 3, Fig. 4). These observations are consistent with reports of high densities of large gadoids, particularly cod, around artificial reefs, utilizing the abundance of shelter and food (Bergström et al. 2013; Støttrup et al. 2014) and decreasing energy consumption (Schwartzbach et al. 2020). The decline of gadoid abundances in 2015 could potentially be a response to fewer crustacean prey species in the area, but could also have other explanations, such as increased predation. For example, harbor seal, *Phoca vitulina* (ICES 2017; Aarts et al. 2019) is currently very abundant in the area, to the extent that they have been suffering from malnutrition (Hårding et al. 2018). Harbor seal predation is estimated by ICES (2017) to have significant stock-level effects on cod in Kattegat, and especially in areas of high seal density, the impacts of seal predation can be pronounced. Still, cod bycatch mortality, alterations in fisheries affecting food sources for cod and failed protection of juvenile habitats may also, in addition to predation, explain lower abundances of cod (Bryhn et al. 2022) and makes it difficult to establish possible causes. Also, the lack of fyke net fishing data from the NTAR during 2007–2014 cannot inform if this decrease in 2015 is just representing the situation for a single year or if it is part of a more long-term declining trend. The mesopredatory fish, rock cook and corkwing wrasse, on the other hand, sustained high abundances in the NTAR throughout the study, presumably benefitting from the artificial reefs, but many other explanations are also plausible.

We observed a decrease in abundance of small decapod crustaceans in the NTAR, but not in the reference area over time, even though the artificial reefs provide suitable habitats for these species. Howarth et al. (2017) reported similar decreases in decapod crustaceans in Scotland in parallel with a strong lobster increase after protection. We may thus explain this decrease by an increased presence of predatory species such as lobster and gadoid fish.

The inverse relationship between the abundance of predators and the abundance of potential prey species, here small decapod crustaceans, in the NTAR (Fig. 5), suggests that the local food web became increasingly top-down regulated over time. No similar relationships occurred in the reference area. Previous studies show that mesopredatory species, i.e. small non-piscivore fish and crabs, have increased along the Swedish west coast, which has been attributed to reductions in large predatory fish, indicating that predators affect the structure and dynamics of entire food webs (Moksnes et al. 2008; Östman et al. 2016). Our study suggests that both large gadoids and lobster have the potential to regulate the occurrences of smaller decapod crustaceans. Thus, the establishment of the NTAR has quite rapidly contributed to a locally re-established top-down regulation, thereby indicating that these measures are useful for restoring ecosystem functions. Similar increases in large predatory fish close to offshore wind farms in Sweden have been reported before (Bergström et al. 2013).

Benthic assemblages benefit in various ways from both primary and secondary artificial reefs, for example by increased species abundance, biomass and diversity compared with the surrounding environment (Langhamer and Wilhelmsson 2009; Granneman and Steele 2015). Even though most of the initial increase in connection with artificial reefs may take place locally among small-bodied and short-lived species, these may attract secondary colonizers and predator species to the reefs as colonization proceeds (Pickering and Whitmarsh 1997; Støttrup et al. 2014). Still, for many species, it is unclear if observed increases are merely due to attraction to the reefs or if also an increased production takes place (Pickering and Whitmarsh 1997; Smith et al. 2016; Schwartzbach et al. 2020), although the support for positive production effects seems to increase (Smith et al. 2016; Roa-Ureta et al. 2019). With regard to the current study, the no-take area might have contributed more to the observed increases in lobster and gadoid fish than the establishment of the artificial reefs, based on an increase in abundance of large individuals over time for both lobster and cod, while at the same time there was a decrease in small decapod crustaceans. This assumption is supported by the fact that lobster abundance also increased at natural reefs inside the NTAR, not only at the artificial reefs.

The establishment of no-take marine reserves has been generally advocated as an ecosystem tool in ecosystem-based management to restore fish populations and marine food webs (Halpern 2003; Fenberg et al. 2012; Moland et al. 2021). No-take reserves are particularly well suited for the management of mixed fisheries and local populations, including many coastal fish assemblages, and can be especially important when adverse ecosystem effects of fishing need to be counteracted (Lester et al. 2009; Sciberras et al. 2013; Berkström et al. 2022). In a wider context, no-take areas are also useful as references for marine environmental management.

Previous work has shown that larger no-take areas can be expected to generate more positive effects in target species (Halpern 2003; Vandeperre et al. 2011; Edgar et al. 2014). In the present study, the no-take areas were relatively small, 1.2–3.2 km^2^, but still demonstrated positive effects on target species for fisheries. A partial explanation to this is that both lobster and gadoid fish can be highly stationary as long as they have access to suitable habitats, so that population densities and individual sizes may increase even within small no-take areas (see also Moland et al. 2013a, b; Øresland and Ulmestrand 2013; Kristensen et al. 2017). The results also indicate that both lobster and local gadoid populations may be managed by small-scale spatial measures such as no-take zones, and that these areas, potentially in combination with artificial reefs, may benefit surrounding areas open to fishing by an elevated productivity, reproductive output and spillover of larvae.

The present study shows that spatially restricted management measures can be effective to support assemblages of fish and large crustaceans, and that positive results can occur rapidly (within 1–2 years). The increased lobster abundance, particularly of large individuals, indicates a direct relationship to decreased fishing pressure and so does the higher abundances of cod and poor cod observed early on in the NTAR. The results, further, indicate that local restoration of large predator populations may also initiate regulatory functions in the food web, as seen by a re-established top-down control in the NTAR. By this, the study exemplifies how local measures can be used to alleviate the impact of human-induced physical and biological pressures related to overfishing and coastal construction and as such constitutes a school example of the principles and potential benefits of marine conservation and restoration. However, the Vinga NTAR also serves as demonstration of potentially achievable environmental conditions for marine ecosystems in an intensively utilized marine region, where the normal situation today is that many populations and habitats are under high pressure from human activities. Continuation of the Vinga NTAR and studies to further follow up on its potential ecosystem-level effects over the long term are needed to see to what extent the responses observed may be further accentuated, and if they can also be manifested in the food web at larger spatial scales. Combining artificial reefs with species/habitat protection is not only important for creating refugia for large predatory species as part of an ecosystem-based management. The measures are also important for facilitating scientific investigations of the importance of reef habitats in coastal environments, as this habitat type still has large knowledge gaps in the northeastern Atlantic and in the Baltic Sea.

## Data Availability

Data can be provided upon reasonable requests to the corresponding author.
